# Supplementary material for: Patterns of Intron Gain and Loss in Fungi
Source: PLoS Biol. 2004 Nov 30;2(12):e422. doi: 10.1371/journal.pbio.0020422 (PMC532390; doi:10.1371/journal.pbio.0020422)
Supplement: Table S1 — Also available at http://genes.mit.edu/NielsenEtAl/. (4.3 MB ZIP). [file pbio.0020422.st001.zip › NielsenEtAl/html/831.html]

AN2317.1.NCU01587.1.MG03164.1.FG06245.1


```
 CLUSTAL W (1.82) Multiple Sequence Alignments - Introns Inserted


Sequence 1: NCU01587.1	136 aa
Sequence 2: FG06245.1	144 aa
Sequence 3: MG03164.1	152 aa
Sequence 4: AN2317.1	161 aa
Alignment Length: 168 aa
Number Identitical Residues: 40 aa
Alignment Score (without introns) 2763


MG03164.1 	-M0SQSG2ATVSQ~ECVTTYNELKLG-----KNIKYIIFKLSDNNKEIVVEEASG--DSD
NCU01587.1	--~----~-----~---------MMS-----RQLRYIIYKLSDDFKEIVIESTSEGATEN
FG06245.1 	--~----~----M0DCITAFNDLKLN-----KKYKFIVYKLSDDYKEIVIDKASE--SRD
AN2317.1  	MS0LASG2VQVQD~ECITAFNNLRMTGGQKGSKPKFIIFKISDDKKQVVVDETSD--DPD
          	     :.    .  .. :: ..  : ..... : ::*::*:**: *::*::.:*     :

MG03164.1 	WENFRNKLVNATVKSPS0GAVGKAPRYAVYDFQYTLASGEGER2NKITFIAWSPDDAGVK
NCU01587.1	YDEFREKLVNAQTKSAS0GAISKGPRYAVYDFEYKLASGEGSR~NKVTFIAWSPDDAGIK
FG06245.1 	WEDFRETLVNATAKSRT0GAVGKGPRYAVYDFEYNLASGDGIR2NKITFIAWSPDDAGIQ
AN2317.1  	YETFLNKLG--DAKDAN~GKP--APRYAVYDVEYDLGGGEGTR2SKIIFISWVPSGTSIN
          	:: * :.*    .*. . *    .*******.:* *..*:* * .*: **:* *..:.::

MG03164.1 	0P-------KMIYAASKDALKRALNGIAHELQANDADDIEYDSVLKTVSKGLA-
NCU01587.1	0S-------KMVYASSKEALKRSLSGIAVELQANEQDDIEYEQIIKTVSKGTAA
FG06245.1 	0P-------KMIYASSKEALKRSLTGIATELQANDTDDIEYDSILKTVSKGLAA
AN2317.1  	0AAHKEQQWSMIYASTREVLKNALN-VVTSIHADDKGDLAWKSILAEVSGGKAK
          	 .: .... .*:**::::.**.:*. :. .::*:: .*: :..::  ** * *
```
